# A Randomized Controlled Trial Comparing Novel Triple-Cuffed Double-Lumen Endobronchial Tubes with Conventional Double-Lumen Endobronchial Tubes for Lung Isolation

**DOI:** 10.3390/jcm9040977

**Published:** 2020-04-01

**Authors:** Namo Kim, Hyo-Jin Byon, Go Eun Kim, Chungon Park, Young Eun Joe, Sung Min Suh, Young Jun Oh

**Affiliations:** 1 Department of Anesthesiology and Pain Medicine, Anesthesia and Pain Research Institute, Yonsei University College of Medicine, 50-1 Yonsei-ro, Seodaemun-gu, Seoul 03722, Korea; namo@yuhs.ac (N.K.); jinoben@yuhs.ac (H.-J.B.); megumi512@naver.com (G.E.K.); joeye@yuhs.ac (Y.E.J.); suh5701@yuhs.ac (S.M.S.); 2 Department of Anesthesiology and Pain Medicine, Gil Medical Center, Gachon University College of Medicine, 21 Namdong-daero 774 beon-gil, Namdong-gu, Incheon 21565, Korea; chungony@gilhospital.com

**Keywords:** airway, double-lumen endobronchial tubes, fiberoptic bronchoscope, one-lung ventilation, thoracic surgery

## Abstract

Placing a double-lumen endobronchial tube (DLT) in an appropriate position to facilitate lung isolation is essential for thoracic procedures. The novel ANKOR DLT is a DLT developed with three cuffs with a newly added carinal cuff designed to prevent further advancement by being blocked by the carina when the cuff is inflated. In this prospective study, the direction and depth of initial placement of ANKOR DLT were compared with those of conventional DLT. Patients undergoing thoracic surgery (*n* = 190) with one-lung ventilation (OLV) were randomly allocated into either left-sided conventional DLT group (*n* = 95) or left-sided ANKOR DLT group (*n* = 95). The direction and depth of DLT position were compared via fiberoptic bronchoscopy (FOB) after endobronchial intubation between the groups. There was no significant difference in the number of right mainstem endobronchial intubations between the two groups (*p* = 0.468). The difference between the initial depth of DLT placement and the target depth confirmed by FOB was significantly lower in the ANKOR DLT group than in the conventional DLT group (1.8 ± 1.8 vs. 12.9 ± 9.7 mm; *p* < 0.001). In conclusion, the ANKOR DLT facilitated its initial positioning at the optimal depth compared to the conventional DLT.

## 1. Introduction

One-lung ventilation is performed to facilitate proper visualization of the surgical field during thoracic surgery or separation of the lungs in an emergent situation [1]. A double-lumen endobronchial tube (DLT) is more commonly used in one-lung ventilation than bronchial blockers with a single-lumen tube, because DLT facilitates more rapid and reliable lung isolation and lung deflation [2]. Positioning a DLT in the appropriate direction and at the appropriate depth is essential for isolating the lungs and preventing complications [3]. However, previous studies report malpositioning requiring subsequent repositioning in approximately 35% to 48% of DLT procedures, even when they are conducted by experienced clinicians [4,5,6]. Endobronchial intubation with a DLT is also more likely to result in airway injury, probably because DLT is stiffer, has a larger external diameter, and is inserted deeply into the mainstem bronchus [7].

A novel triple-cuffed ANKOR DLT (Insung Medical, Wonjou, Republic of Korea) was recently developed to facilitate proper DLT positioning via the addition of a carinal cuff between the bronchial cuff and the tracheal lumen [8]. The ANKOR DLT may facilitate superior DLT positioning and reduce tracheobronchial injury, but its effectiveness remains to be conclusively elucidated. The primary endpoint of this study was to compare ANKOR DLT and conventional DLT for initial DLT positioning in patients who underwent thoracic surgery with one-lung ventilation. The secondary endpoint was to compare the tracheobronchial injury immediately after endobronchial intubation in both groups.

## 2. Materials and Methods 

### 2.1. Study Population

**Figure 1:**
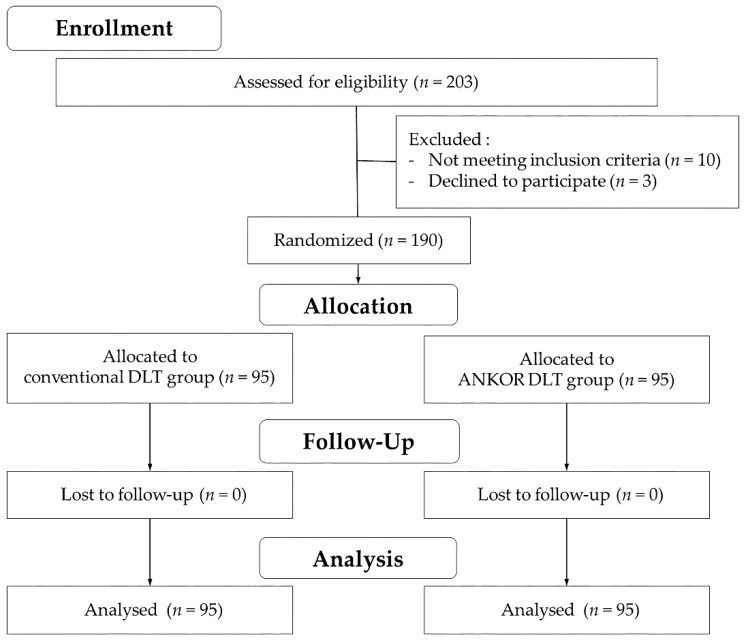
CONSORT flow diagram. DLT, double-lumen endobronchial tube; FOB, fiberoptic bronchoscopy.

The present study was approved by the Institutional Review Board of Severance Hospital, Yonsei University Health System, Seoul, Republic of Korea (No. 4-2018-0698) and registered at clinicaltrials.gov (NCT03782090). After obtaining written informed consent, 190 patients requiring one-lung ventilation with a left-sided DLT for thoracic surgery were randomly allocated to either a conventional DLT group (*n* = 95) or an ANKOR DLT group (*n* = 95) via a computerized randomization table (Figure 1). Inclusion criteria were an American Society of Anesthesiologists physical status of I, II, or III, and age between 20 and 85 years. Exclusion criteria were the presence of intraluminal surgical lesions in the left or right bronchus, anatomic anomalies in the tracheobronchial tree such as tracheomalacia or tracheal bronchus, expected difficult airway (neck extension <35°, mandibular-hyoid distance <6.0 cm, and sternomental distance <12.5 cm), a body mass index >30 kg/m^2^, upper respiratory infection, a history of thoracic surgery, any blood coagulation disorder, and the requirement of an emergency operation.

### 2.2. Anesthetic Management and Procedures

Standard monitoring, including pulse oximetry, electrocardiography, sphygmomanometry, and end-tidal carbon dioxide evaluation, was performed when patients arrived at the operating room. Anesthesia was induced with 1.0 to 1.5 mg/kg propofol, 0.5 to 1.0 µg/kg remifentanil, and 2.0 to 4.0 vol% sevoflurane inhalation, and 0.6 mg/kg rocuronium bromide was administered to facilitate intubation. All lung isolations were performed using either an ANKOR DLT or a conventional DLT by the same investigator who specializes in thoracic anesthesia. The sizes of conventional DLTs (32, 35, 37 and 39 Fr) and ANKOR DLTs (33, 35, 37, and 39 Fr) were chosen based on the inner diameter of the left main bronchus as determined via coronal view of chest computed tomography (< 11 mm, 32 or 33 Fr; 11 ≤ and < 13 mm, 35 Fr; 13 ≤ and < 15 mm, 37 Fr; 15 mm ≤, 39 Fr).

In the conventional DLT group, a polyvinyl chloride Shiley^®^ DLT (Covidien, Mansfield, MA, USA) was used to achieve lung isolation. The DLT was inserted into the glottis via direct laryngoscopy, and the stylet was removed from the DLT after the bronchial cuff had passed the vocal cord. The DLT was then rotated 90° counterclockwise and advanced to the depth calculated using the formula 12.5 + 0.1 × height in cm [9]. For the initial evaluation of DLT position, both sides of the chest were auscultated and chest wall movements were observed before and after selective clamping of the tracheal and bronchial lumens. Compliance of the lungs was assessed by comparing the peak inspiratory pressure of the right and left lungs before and after clamping the tracheal and bronchial lumens. Thereafter, the depth of the DLT was adjusted by checking the compliance of both the lungs manually [10,11].

**Figure 2:**
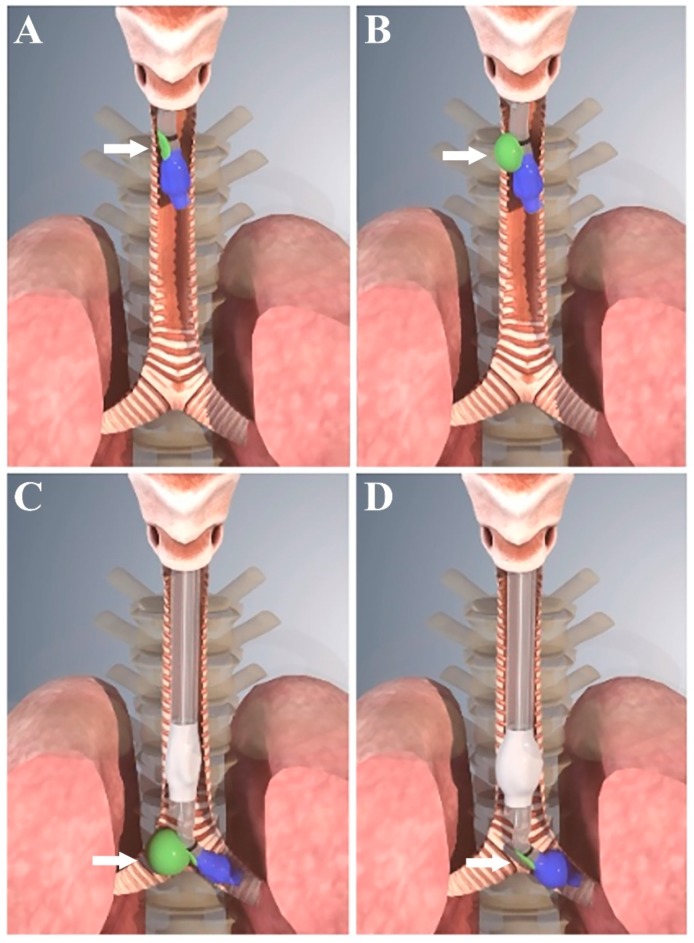
Schematic illustration showing the use of ANKOR double-lumen endobronchial tube (DLT). (**A**) The carinal cuff (green color, indicated by the white arrows) of the tube passed beyond the vocal cord, and the ANKOR DLT was turned to the left; (**B**) The carinal cuff was inflated with 5 to 7 mL of air; (**C**) The ANKOR DLT was advanced until the inflated carinal cuff is functionally anchored at the keel-shaped carinal ridge; and (**D**) After the deflation of the carinal cuff, the tracheal cuff (white color) and the bronchial cuff (blue color) of the tube were inflated with air.

The structure of ANKOR DLT is similar to that of conventional polyvinyl chloride DLT, but is made of 100% silicone. In addition, ANKOR DLT has a carinal cuff located at a point between the distal opening of the tracheal lumen and the proximal margin of the bronchial cuff. After the carinal cuff of the ANKOR DLT passes the vocal cord, the tube is rotated 90° counterclockwise [8], followed by inflation of the carinal cuff with 5 to 8 mL of air (33 Fr, 5 mL; 35 Fr, 6 mL; 37 Fr, 7 mL; 39 Fr, 8 mL). It is then advanced alongside the tracheobronchial tree until the inflated carinal cuff is blocked by the carina and can no longer be advanced, at which point the ANKOR DLT is positioned at the depth required for lung isolation. Lastly, the carinal cuff is deflated, and the bronchial and tracheal cuffs are inflated (Figure 2). In the present study mechanical ventilation was initiated using auto-flow pressure-controlled ventilation mode (Primus i ventilator; Dräger^TM^ Medical, Lübeck, Germany) in both groups.

### 2.3. Direction and Depth of DLT

The primary outcomes were the direction and depth of DLT placement on the first attempt. After initial assessment using a stethoscope, the direction of endobronchial intubation was evaluated via fiberoptic bronchoscopy (FOB), which was performed by another investigator who specializes in thoracic anesthesia. If the DLT was inserted into the right mainstem bronchus, the patient was excluded from further assessment in measuring the depth difference of DLT placement and the injuries of tracheobronchial tree. After endobronchial intubation was achieved into the left mainstem bronchus, the depth difference between initial DLT placement and target depth was measured. The target depth of the DLT position was where the proximal margin of the inflated bronchial cuff was immediately below the tracheal carina, demonstrating a clear view of the left upper and left lower lobe bronchus through the bronchial lumen as confirmed by FOB in both groups. The depth of DLT placement was measured at the teeth before and after repositioning the DLT to the target depth. The depth difference of DLT placement was compared in mean difference, and numbers of patients in each following section of depth difference: (1) ≤5 mm; (2) 6–10 mm; (3) 11–20 mm; (4) >20 mm.

### 2.4. Time Taken to Achieve Lung Isolation

The time taken to achieve initial lung isolation was defined as the time from the passage of the distal tip of the DLT endobronchial lumen beyond the vocal cords to the initial accomplishment of left mainstem endobronchial intubation. The time taken to confirm DLT depth via FOB was defined as the time from the passage of the distal tip of the bronchoscope through the proximal opening of the DLT endobronchial lumen to the point when the depth of the DLT position was confirmed.

### 2.5. Tracheobronchial Tree Injury Measurements

After confirming the direction and depth of the DLT position, the bronchial and tracheal cuffs were deflated and the DLT was withdrawn to enable the evaluation of tracheobronchial injury via FOB observation through the endobronchial lumen. The level of injury was graded on a previously described five-point scale (0, clear; 1, a few petechiae; 2, coalesced petechiae; 3, erosion; 4, more severe injury) [12].

### 2.6. Statistical Analysis

The sample size required was estimated based on the results of a previous study that reported a 63% success rate with regard to placing the DLT in the optimal position [13]. We estimated that random assignment of subjects to two groups of *n* = 95 was required to detect a difference of 20% in the success rate of placing a DLT in the optimal position, with 80% power at the 5% significance level, allowing for a maximum drop-out rate of 10%. Statistical analyses were performed using SPSS 23 (SPSS Inc., Chicago, IL, USA). Normality of datasets was assessed via the Kolmogorov–Smirnov test. All data are expressed as means ± the standard deviation, ranges, numbers and percentages, or medians and interquartile ranges as indicated. Data of the two groups were compared using the chi-square test, Mann–Whitney *U*-test, and Student’s *t*-test as appropriate. *p* < 0.05 was deemed to indicate statistical significance.

## 3. Results

A total of 190 patients undergoing thoracic surgery were included in the study, and there were no desaturation events during the experimental period and all thoracic surgery was successfully performed without any complications. There were no statistically significant differences in patient characteristics and the selected size of DLT between the two groups (Table 1).

The number of intubations into the right mainstem bronchus on the first attempt did not differ significantly between the two groups (*p* = 0.468) (Table 2). The mean difference in the depth of DLT positioning between initial placement and FOB confirmation was significantly lower in the ANKOR DLT group than in the conventional DLT group (*p* < 0.001). The incidence of less than 5 mm of difference between DLT placement depth and the target depth was significantly higher in the ANKOR DLT group than in the conventional DLT group (*p* < 0.001). There was no incidence of a difference of greater than 10 mm between DLT placement depth and the target depth in the ANKOR DLT group. Conversely, in the conventional DLT group, the difference was >10 mm in 52.4% of patients and >20 mm in 21.4% of patients. The time taken for FOB confirmation did not differ significantly between the two groups, but the time taken for initial lung isolation was significantly longer in the conventional DLT group (*p* = 0.014).

The overall tracheobronchial injury score showed significant difference between the two groups (*p* = 0.035) (Table 3). There were no cases of level 4 injury in either group, and there were no significant differences in the incidences of level 1 or level 2 tracheobronchial injuries between the two groups; however, the incidence of level 3 tracheobronchial injury was significantly higher in the conventional DLT group (*p* = 0.008). Injuries of tracheobronchial tree were mainly observed on the left side of tracheal wall, carinal ridge, and left mainstem bronchus. However, none of the patients in either group were observed tracheobronchial injury on the right side of the tracheal wall.

## 4. Discussion

In the current study, ANKOR DLT tended to be placed in a more appropriate position that was closer to the target depth than conventional DLT, while there was no significant difference in the incidence of mis-directional endobronchial intubation between the two groups. ANKOR DLT placement was also less time consuming and less traumatic than that of conventional DLT.

Lung isolation for thoracic surgery is conducted by thoracic anesthesiologists as well as non-thoracic anesthesiologists or trainees with limited experience in thoracic surgery. Although FOB has facilitated improved visualization of the tracheobronchial anatomy and is considered the gold standard for confirming the position of the DLT [14], in a previous study, 39% of anesthesiologists with limited thoracic anesthesia experience were unable to achieve lung isolation successfully irrespective of the type of device used, due to poor knowledge of endoscopic bronchial anatomy [5]. Devices such as VivaSight DLT (ETView Ltd., Misgav, Israel), with a built-in videoscope that makes it possible to directly examine the interior of the bronchus, have been developed to achieve effective lung isolation [15]; however, there is a controversy suggesting that even VivaSight DLT does not reduce the use of FOB [16].

The inflated carinal cuff of the ANKOR DLT that expands toward the right side of the main body of the DLT is subject to being captured by the carinal ridge as the tube is advanced alongside the tracheobronchial tree. The point at which the ANKOR DLT is unable to advance further is considered the proper depth for lung isolation, and the inflated carinal cuff is assumed to affect the direction of ANKOR DLT insertion into an intended left mainstem bronchus. Notably, however, in the current study, the incidence of ANKOR DLT insertion into the left mainstem bronchus on the first attempt was comparable to that associated with conventional DLT. These results are consistent with those of a previous study in which the incidence of right endobronchial misplacement during the insertion of a left-sided DLT was 8% using the blind technique [17].

Misplacement of a DLT is deemed to have occurred when the tube needs to be repositioned by more than 5 mm [13,18]. Compared with conventional DLT, ANKOR DLT were more optimally positioned before FOB guidance. The results of the present study suggest that lung isolation by using ANKOR DLT can be accomplished regardless of using FOB. Using ANKOR DLTs can reduce the need for multiple attempts of DLT placement for lung isolation, which entails inevitable risks of airway trauma, hypoxemia, and hypercapnia, particularly when non-thoracic anesthesiologists or trainees are conducting the procedure [19]. It is also notable that given this feature of ANKOR DLT, their use may be considered in situations in which FOB is not feasible, such as in cases of massive pulmonary secretion or bleeding. In a previous case report, an ANKOR DLT was successfully placed to perform lung isolation in a lung transplant patient who exhibited massive secretion due to idiopathic pulmonary fibrosis [8]. Furthermore, given that there are substantial costs associated with the use, maintenance, and repair of FOB equipment, the use of ANKOR DLT may have some cost/benefit advantages with respect to reducing the use of FOB resources in developing countries or small-volume institutions [19]. Taken together, the advantage of using ANKOR DLT is that the accuracy can be increased in positioning the DLT by simply adding the carinal cuff located at a point between the distal opening of the tracheal lumen and the proximal margin of the bronchial cuff.

Lung isolation with ANKOR DLT was less time consuming than those with conventional DLT, although the mean difference in the time taken for the confirmation of lung isolation with FOB was comparable in the two groups. The reduced intubation time is thought to be due to the structure of ANKOR DLT, which helps the investigator determine DLT depth without hesitation, though it does take more time to manipulate the carinal cuff. However, under circumstances in which the depth and position can be confirmed by FOB, the mean 10-second difference in the current trial may be of low clinical relevance.

In this study, most of the injuries occurred on the left side of tracheal wall, the carinal ridge, left mainstem bronchus and there was little injury to the right side of the tracheal wall. Taking into consideration the location of the injuries, the tracheobronchial injury with DLT placement were more likely caused by the angled tip of DLT during endobronchial intubation and not by the inflated carinal cuff. Interestingly, erosion was less prominent when using the ANKOR DLT, whereas mild injuries, such as petechiae, were observed at similar rates in both groups. Considering that the structure of ANKOR DLT is similar to that of the conventional DLT, less injuries with the ANKOR DLT may be attributed to material of the ANKOR DLT, which is made of silicone making them softer and more supple than the conventional DLT, which is made of polyvinyl chloride [20].

The present study had following limitations. First, by necessity the investigator who performed DLT placement was not blinded to the type of DLT used in this study. However, DLT placement and FOB confirmation were performed by the independent investigators with the standardized study protocol. Second, all lung isolations in the current study were conducted by thoracic anesthesiologists and the experience and skill of the physician who performed endobronchial intubations using ANKOR DLT and conventional DLT may also have affected the results of the study [5]. Accordingly, the same results cannot be guaranteed when endobronchial intubation is performed by a physician with a different level of skill or experience. Third, although there were no cases of desaturation or the surgeon’s complaint regarding the lung collapsibility, neither the quality of lung isolation nor frequency of malpositions were investigated during the surgery. Finally, because the use of the carinal cuff was limited to initial placement, we did not investigate overall post-operative complications such as coughing, hoarseness and sore throat as well as the extent of tracheobronchial injury at the end of the surgery. Further investigations evaluating the effectiveness of ANKOR DLT in a range of clinical circumstances are warranted.

## 5. Conclusions

In conclusion, the ANKOR DLT was able to guide its own positioning to the appropriate depth better than conventional DLT. Moreover, its use was associated with less trauma of the tracheobronchial tree than the use of conventional DLT.

## Figures and Tables

**Table 1:** Patients’ characteristics.

Parameters	Conventional DLT (*n* = 95)	ANKOR DLT (*n* = 95)	*p*-Value
Age (years)	58.1 ± 11.7	60.4 ± 11.6	0.340
Height (cm)	163.6 ± 8.9	160.9 ± 8.5	0.050
Weight (kg)	65.9 ± 10.5	63.5 ± 10.5	0.093
Sex (female/male)	45/50	54/41	0.148
BMI (kg/m^2^)	24.6 ± 3.2	24.5 ± 3.5	0.845
Medical history, *n* (%)			
Hypertension	32 (33.7%)	35 (36.8%)	0.649
Diabetes mellitus	19 (20.0%)	18 (18.9%)	0.855
Chronic obstructive pulmonary disease	1 (1.1%)	2 (2.1%)	1.000
Emphysema	1 (1.1%)	1 (1.1%)	1.000
Bronchiectasis	1 (1.1%)	1 (1.1%)	1.000
Interstitial lung disease	0 (0%)	3 (3.2%)	0.246
Diameter of left mainstem bronchus (mm)	12.6 ± 1.1	12.5 ± 1.1	0.352
DLT size, *n* (%)			0.831
32 or 33 Fr	2 (2.1%)	2 (2.1%)	1.000
35 Fr	51 (53.7%)	56 (59.0%)	0.465
37 Fr	42 (44.2%)	37 (38.9%)	0.462
39 Fr	0 (0%)	0 (0%)	N/A

Data are presented as number of patients (%) or mean ± standard deviation. BMI, body mass index; DLT, double-lumen endobronchial tube; N/A, not applicable.

**Table 2:** Direction of left-sided DLT endobronchial intubation, depth difference between initial DLT positioning and target depth confirmed by FOB, and time for DLT positioning and FOB confirmation.

Parameters	Conventional DLT (*n* = 95)	ANKOR DLT (*n* = 95)	*p*-Value
Intubation into the right mainstem bronchus, *n* (%)	11 (11.6%)	8 (8.4%)	0.468
Intubation into the left mainstem bronchus, *n* (%)	84 (88.4%)	87 (91.6%)	
Depth difference			
Mean (mm)	12.9 ± 9.7	1.8 ± 1.8	<0.001
Numbers of patients at each section, *n* (%)			<0.001
≤ 5 mm	26 (31.0%)	83 (95.4%)	<0.001
6–10 mm	14 (16.6%)	4 (4.6%)	0.012
11–20 mm	26 (31.0%)	0 (0%)	<0.001
> 20 mm	18 (21.4%)	0 (0%)	<0.001
Time for initial placement (sec)	69.7 ± 33.9	59.9 ±12.1	0.014
Time for FOB confirmation (sec)	25.2 ± 12.0	24.7 ± 10.3	0.748

Data are presented as number of patients (%) or mean ± SD. DLT, double-lumen endobronchial tube; FOB, fiberoptic bronchoscopy.

**Table 3:** The injury of tracheobronchial tree after initial DLT placement into the left mainstem bronchus.

Injury score	Conventional DLT (*n* = 84)	ANKOR DLT(*n* = 87)	*p*-Value
Injury score, *n* (%)			0.035
0, Clear	2 (2.4%)	1 (1.2%)	0.616
1, A few petechiae	37 (44.0%)	43 (49.4%)	0.481
2, Coalesced petechiae	26 (31.0%)	36 (41.4%)	0.128
3, Erosion	19 (22.6%)	7 (8.0%)	0.008
4, More severe injury	0 (0%)	0 (0%)	N/A

Data are presented as number of patients (%). DLT, double-lumen endobronchial tube. 0, clear; 1, a few petechiae; 2, coalesced petechiae; 3, erosion; 4, more severe injury; N/A, not applicable.
